# Pretreatment with H_2_O_2_ Alleviates the Negative Impacts of NaCl Stress on Seed Germination of Tartary Buckwheat (*Fagopyrum tataricum*)

**DOI:** 10.3390/plants10091784

**Published:** 2021-08-27

**Authors:** Xin Yao, Meiliang Zhou, Jingjun Ruan, Yan Peng, Hao Yang, Yong Tang, Anjing Gao, Jianping Cheng

**Affiliations:** 1 College of Agronomy, Guizhou University, Guiyang 550000, China; yaoxin123@hotmail.com (X.Y.); pengyan123@hotmail.com (Y.P.); yanghao123@hotmail.com (H.Y.); tangyong114@hotmail.com (Y.T.); gaoanjing123@hotmail.com (A.G.); 2 Institute of Crop Science, Chinese Academy of Agriculture Science, Beijing 100000, China; zhoumeiliang@caas.cn

**Keywords:** *F. tataricum*, germination, hydrogen peroxide, NaCl, seed soaking

## Abstract

Soil salinization is one of the main abiotic stress factors impacting the growth of crops and the agricultural industry today. Thus, we aimed to investigate the effects of H_2_O_2_ pretreatment on seed germination in Tartary buckwheat (*Fagopyrum tataricum*) seeds under salt stress and to evaluate this species’ salt tolerance. Through the preliminary experiment, this study used 50 mmol L^−1^ NaCl solution to induce seed stress. After soaking for 12 h in different H_2_O_2_ concentrations, seeds were laid in Petri dishes with 50 mmol L^−1^ NaCl for seven days and the germination parameters and physiological indicators were measured to screen the optimal H_2_O_2_ pretreatment concentration and the salt tolerance index. Our results indicated that pretreatment with 5–10 mmol L^−1^ H_2_O_2_ was most effective in alleviating NaCl’s impacts on the seeds’ germination parameters. Furthermore, the growth and material accumulation of seedlings was promoted; catalase, superoxide dismutase activity, and proline content were enhanced; and malondialdehyde content was reduced. Principal component analysis and stepwise regression revealed six key indicators that had a significant impact on the salt tolerance characteristics of *F. tataricum*, namely, germination potential, shoot fresh weight, root surface area, root average diameter, catalase activity, and superoxide dismutase activity.

## 1. Introduction

Soil salinization is one of the main abiotic stress factors, which affects crop morphology, physiology, biochemistry, and gene expression. Furthermore, it restricts the planting and growth of certain varietals and limits the potential for agricultural development. The area impacted by soil salinization is expanding worldwide [1]. Saline soils in China represent approximately 3600 × 104 ha, which accounts for about 25% of the cultivated land area [2]. Studies have found that exposure to salt stress inhibits most plant growth parameters to varying degrees [3,4]. Therefore, screening salt-tolerant varieties and studying the tolerance of crops to salt stress are effective methods for improving their adaptability. Furthermore, it is crucial to examine the growth patterns of crops under suitable salt concentrations. Hydrogen peroxide (H_2_O_2_) is a relatively stable, free-diffusing, and fairly long-lived active oxygen compound [5,6]. When crops are under various types of stress, the application of exogenous H_2_O_2_ can alleviate the stress and reduce crop damage. For example, pretreatment with exogenous H_2_O_2_ can increase the active enzyme content of rice under drought stress [7], reduce salt-induced damage to wheat roots [8], and improve the cold resistance and cell viability of rape seedlings [9].

Buckwheat may refer to a variety of dicotyledonous plants in the genus *Fagopyrum* within the family *Polygonaceae*. They can be annual or perennial, and the plants are used for food and animal feed [10]. In China, buckwheat is divided into three major cultivated species: Tartary buckwheat (*F. tataricum*), sweet buckwheat (*F. esculentum*) and golden buckwheat (*F. cymosum*), and the rest are wild species [11]. *F. tataricum* is a cold-tolerant crop suitable for cultivation in high-altitude mountainous areas with short frost-free periods, such as southwestern China [12]. It has a high nutritional value, important health benefits, and contains starch, protein, vitamins, mineral elements and other nutrients. *F. tataricum* is also rich in flavonoids and other biologically active substances, which can help mitigate diabetes, prevent cardiovascular sclerosis, and regulate high blood pressure [13,14,15].

*F. tataricum* frequently grows in poor quality soil including soils with increased salinity and H_2_O_2_ can alleviate the negative impacts of salt stress on seed germination. At present, there are few studies that investigate the effect of H_2_O_2_ seed soaking on the germination characteristics of *F. tataricum* seeds under salt stress [16,17]. We soaked *F. tataricum* seeds in different concentrations of H_2_O_2_ to examine the germination characteristics, seedling growth, antioxidant enzyme activity, membrane lipid peroxidation, and osmotic adjustment substances present in seeds under salt stress. A principal component analysis, stepwise regression analysis, and other methods were used to evaluate the salt tolerance of *F. tataricum*. Our aim was to investigate the effects of H_2_O_2_ pretreatment on alleviating the inhibition of *F. tataricum* seeds under salt stress and test the relationship between salt tolerance indicators and pretreating seeds with H_2_O_2_. Through this study, we hope to provide the theoretical basis for a treatment to improve the salt tolerance of *F. tataricum* seeds.

## 2. Results

### 2.1. The Effects of Different NaCl Concentrations on the Seed Germination

The germination potential (GP), germination rate (GR), germination index (GI), vigor index (VI) and shoot length (SL) of *F. tataricum* seeds all decreased as the concentration of NaCl treatment increased (Table 1). VI and SL decreased significantly under 50 mmol L^−1^ NaCl compared to those under 0 mmol L^−1^ NaCl (*p* < 0.05); and GI, VI, and SL were significantly inhibited under the 100 mmol L^−1^ NaCl treatment (*p* < 0.05) (Table 1). However, all five germination indices further decreased under the 150 and 200 mmol L^−1^ NaCl treatments (*p* < 0.05) (Table 1). As the concentration of NaCl treatments was increased from 0 to 50, 100, 150 and 200 mmol L^−1^, VI decreased by 77.0%, 88.2%, 91.9%, and 95.2%, respectively; and SL decreased by 75.6%, 82.4%, 85.7%, and 89.0%, respectively (Table 1). In summary, 50 mmol L^−1^ NaCl significantly reduces GP, GR and GI, and significantly inhibits VI and SL by more than 75.0% (Table 1). Therefore, going forward, this study used 50 mmol L^−1^ NaCl solution to induce seed stress (Table 1).

### 2.2. The Effects of H_2_O_2_ Pretreatment and NaCl Stress on the Seed Germination

**Figure 1:**
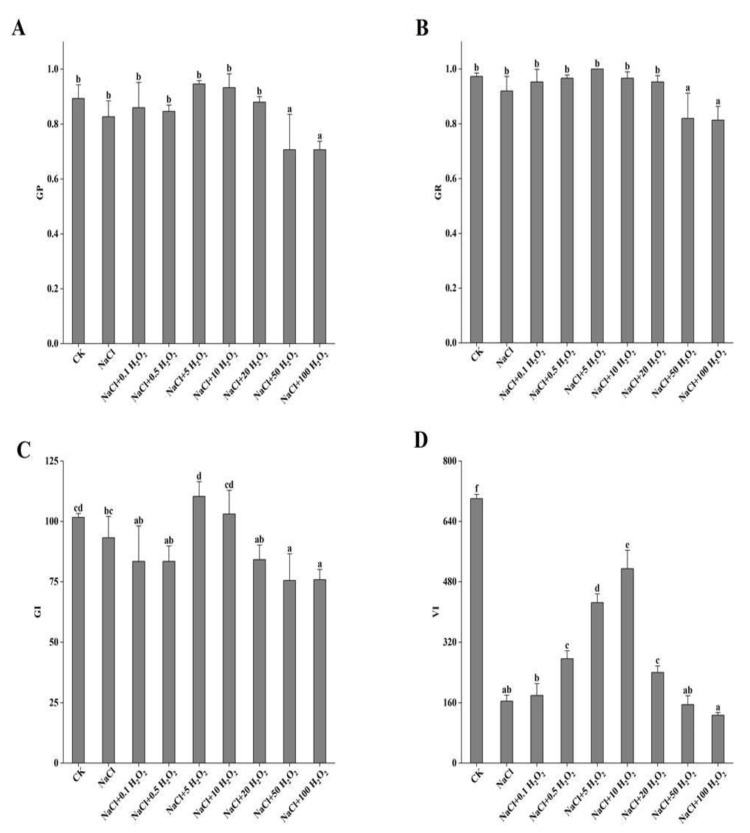
Effect of H_2_O_2_ pretreatment on the GP (**A**), GR (**B**), GI (**C**), and VI (**D**) of *F. tataricum* seeds under NaCl stress. CK, control treatment (only water); NaCl, 50 mmol L^−1^ NaCl treatment; NaCl + xxx H_2_O_2_, treatments of standard NaCl + respective H_2_O_2_ concentration; GP, germination potential; GR, germination rate; GI, germination index; VI, vigor index. Different lowercase letters on each column indicate significant differences between treatments at *p* < 0.05.

The 50 mmol L^−^^1^ NaCl treatment had no significant inhibitory effect on GP, GR, and GI of *F. tataricum* seed (*p <* 0.05) (Figure 1A–C); however, it did significantly reduce their VI (*p <* 0.05), which was 76.6% lower than the CK (Figure 1D). Under NaCl stress, treatments of increasing H_2_O_2_ concentration caused an initial increase and then a decrease in the GP, GR, GI, and VI of *F. tataricum* seeds (Figure 1A–D). The GP, GR, and GI of seeds reached their maximum values under the treatment of NaCl + 5 mmol L^−^^1^ H_2_O_2_; increasing by 14.5%, 8.7%, and 18.4%, respectively, compared to those of seeds treated with NaCl alone (Figure 1A–C). VI reached its maximum value under NaCl + 10 mmol L^−^^1^ H_2_O_2_, which was 3.1 times higher than that under NaCl treatment alone (Figure 1D). Under the treatment of NaCl + 100 mmol L^−^^1^ H_2_O_2_, the GP, GR, GI, and VI all noticeably decreased compared to their values under the CK treatment, and NaCl alone (Figure 1A–D). These results suggest that pretreatment with H_2_O_2_ could alleviate the inhibitory effects that *F. tataricum* seeds experience under salt stress.

### 2.3. The Effects of H_2_O_2_ Pretreatment and NaCl Stress on Shoot Length and Fresh Weight

**Figure 2:**
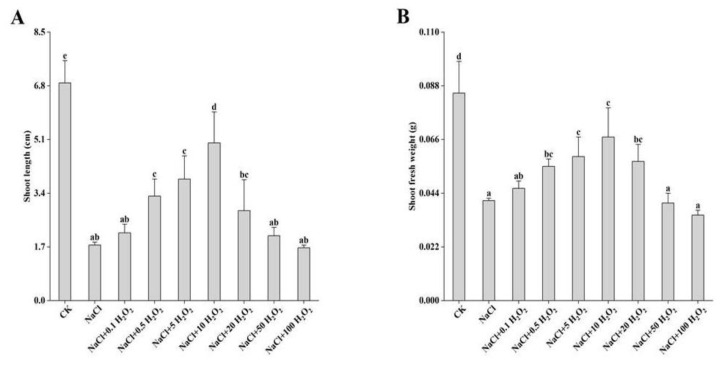
Effect of H_2_O_2_ on SL (**A**) and SFW (**B**) of *F. tataricum* seedlings under NaCl stress. CK, control treatment (only water); NaCl, 50 mmol L^−1^ NaCl treatment; NaCl + xxx H_2_O_2_, treatments of standard NaCl + respective H_2_O_2_ concentration; SL, shoot length (cm); SFW, shoot fresh weight (g). Different lowercase letters on each column indicate significant differences between treatments at *p* < 0.05.

Compared with the measurements in the CK treatment, shoot length (SL) and shoot fresh weight (SFW) decreased significantly under all other treatments (*p <* 0.05) (Figure 2A,B). In the NaCl treatment, SL and SFW of *F. tataricum* seedlings reduced significantly to 25.5% and 48.2% of the control values, respectively (Figure 2A,B). Excluding the control treatment, SL and SFW first increased and then decreased with increasing H_2_O_2_ concentration, reaching their highest values at 0.5–10 mmol L^−^^1^ H_2_O_2_ (Figure 2A,B). Under the 10 mmol L^−^^1^ H_2_O_2_ treatment, SL was 2.8 times higher than that of the NaCl treatment (Figure 2A); while SFW was 1.6 times higher (Figure 2B). However, they both reached their lowest values under the 100 mmol L^−^^1^ H_2_O_2_ treatment (Figure 2A,B).

### 2.4. The Effects of H_2_O_2_ Pretreatment and NaCl Stress on Root Growth

The NaCl treatment had a significant effect on the root growth of *F. tataricum* seedlings compared to that of the control treatment (Table 2). Root length (RL) and Root surface area (RSA) were significantly reduced (*p <* 0.05) by 36.3% and 38.9%, respectively (Table 2); Root average diameter (RAD) and Root volume (RV) were significantly reduced by 4.1% and 35.7%, respectively (Table 2); whereas root fresh weight (RFW) increased significantly by 35.7% (Table 2). Excluding the control treatment, RL, RSA, RAD, RV, and RFW all increased and then decreased as H_2_O_2_ concentration increased (Table 2). Under the NaCl + 0.5 mmol L^−^^1^ H_2_O_2_ treatment, RL, RSA, and RFW reached their maximum values at 1.6, 1.9, and 1.4 times higher than those in the NaCl treatment (Table 2). RAD and RV reached their maximum values under the NaCl + 10 mmol L^−^^1^ H_2_O_2_ treatment, which were 7.3 and 3.4 times those of the NaCl treatment, respectively (Table 2). The RSA of *F. tataricum* seedlings decreased significantly at H_2_O_2_ concentrations of 5–100 mmol L^−^^1^, while the RAD increased significantly (Table 2). These results suggest that pretreatment with H_2_O_2_ can generally alleviate NaCl’s inhibitory effect on the root growth of *F. tataricum* seedlings; however, its effect on RSA was certainly different.

### 2.5. The Effects of H_2_O_2_ Pretreatment and NaCl Stress on Antioxidant Enzyme Activity

**Figure 3:**
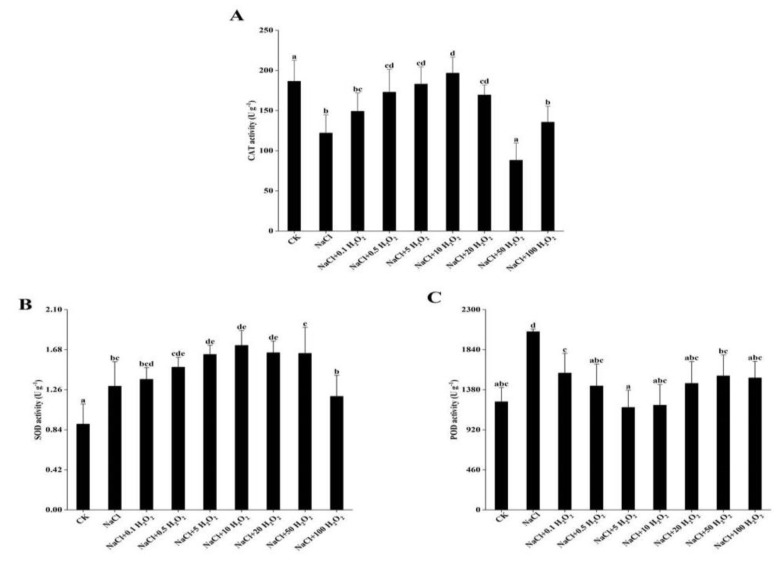
Effect of H_2_O_2_ pretreatment on the activity of CAT (**A**), SOD (**B**), and POD (**C**) in *F. tataricum* seedlings under NaCl stress. CK, control treatment (only water); NaCl, 50 mmol L^−1^ NaCl treatment; NaCl + xxx H_2_O_2_, treatments of standard NaCl + respective H_2_O_2_ concentration; CAT, catalase (U g^−1^); SOD, superoxide dismutase (U g^−1^); POD, peroxidase (U g^−1^). Different lowercase letters on each column indicate significant differences between treatments at *p* < 0.05.

Excluding the CK treatment, CAT and SOD first increased and then decreased in response to increasing H_2_O_2_ concentrations; whereas POD first decreased and then increased (Figure 3A–C). Under the NaCl + 10 mmol L^−^^1^ H_2_O_2_ treatment, the activity of CAT and SOD in *F. tataricum* seedlings reached maximums at 196.6 and 1.7 U g^−1^, respectively (Figure 3A,B). POD activity reached its minimum of 1177.0 U g^−1^ under the treatment of NaCl + 5 mmol L^−^^1^ H_2_O_2_ (Figure 3C).

The SOD and POD activities increased significantly under NaCl stress in *F. tataricum* seedlings (*p <* 0.05) (Figure 3B, C). SOD activity increased significantly in all treatments when compared to that of the control; specifically, by 47.8% in the NaCl + 10 mmol L^−^^1^ H_2_O_2_ treatment (Figure 3B). POD activity in most treatments was higher than in the control, except for that of the NaCl + 5 mmol L^−^^1^ H_2_O_2_ and NaCl + 10 mmol L^−^^1^ H_2_O_2_ treatments (Figure 3C).

The CAT activity was significantly inhibited in *F. tataricum* seedlings under NaCl stress (*p <* 0.05) (Figure 3A). At low (0.1–0.5 mmol L^−^^1^) and high (50–100 mmol L^−^^1^) H_2_O_2_ concentrations, the CAT activity was significantly reduced (Figure 3A). It reached its lowest activity level, at 55.2% below the maximum, under the NaCl + 50 mmol L^−^^1^ H_2_O_2_ treatment (Figure 3A).

### 2.6. The Effects of H_2_O_2_ Pretreatment and NaCl Stress on MDA and Pro Contents

**Figure 4:**
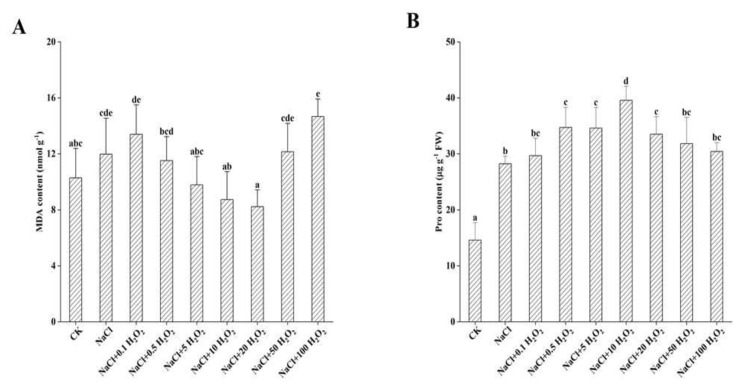
Effect of H_2_O_2_ on MDA (**A**) and Pro (**B**) contents of *F. tataricum* seedlings under NaCl stress. CK, control treatment (only water); NaCl, 50 mmol L^−1^ NaCl treatment; NaCl + xxx H_2_O_2_, treatments of standard NaCl + respective H_2_O_2_ concentration; MDA, malondialdehyde (nmol g^−1^); Pro, proline (µg g^−1^ FW). Different lowercase letters on each column indicate significant differences between treatments at *p* < 0.05.

The MDA content of *F. tataricum* seedlings under the NaCl-only treatment increased significantly by 16.5% (*p <* 0.05) compared to that of the control treatment (Figure 4A). Under the treatments of increasing H_2_O_2_ concentration, MDA content generally decreased first and then increased (Figure 4A). Its minimum value was 8.2 nmol g^−1^ under the NaCl + 20 mmol L^−^^1^ H_2_O_2_ treatment, which was 68.7% of the MDA value in the NaCl treatment (Figure 4A). The MDA content increased significantly under the NaCl + 50 mmol L^−^^1^ and NaCl + 100 mmol L^−^^1^ H_2_O_2_ treatments, indicating that H_2_O_2_ pretreatment significantly reduced the effects of NaCl stress on *F. tataricum* seedlings (Figure 4A). These results indicate that soaking seeds in different H_2_O_2_ concentrations can have varying effects on MDA content.

The Pro content of *F. tataricum* seedlings in each treatment was significantly higher than that of the control treatment (*p <* 0.05) (Figure 4B). It first increased and then decreased with increasing H_2_O_2_ concentration (Figure 4B); reaching its peak under the NaCl + 10 mmol L^−^^1^ H_2_O_2_ treatment which was 2.7 and 1.4 times that of the CK and NaCl treatments, respectively (Figure 4B). Thereafter, further increases in H_2_O_2_ concentration caused a significant decrease in seedling Pro content (Figure 4B); with Pro content of the NaCl + 100 mmol L^−^^1^ H_2_O_2_ treatment decreasing by 23.1% compared to that of the NaCl + 10 mmol L^−^^1^ H_2_O_2_ treatment (Figure 4B). These results indicate that H_2_O_2_ pretreatment increased the content of osmotic adjustment substances in *F. tataricum* seedlings under NaCl stress to a certain extent.

### 2.7. Hierarchical Clustering and Correlation Analysis

**Figure 5:**
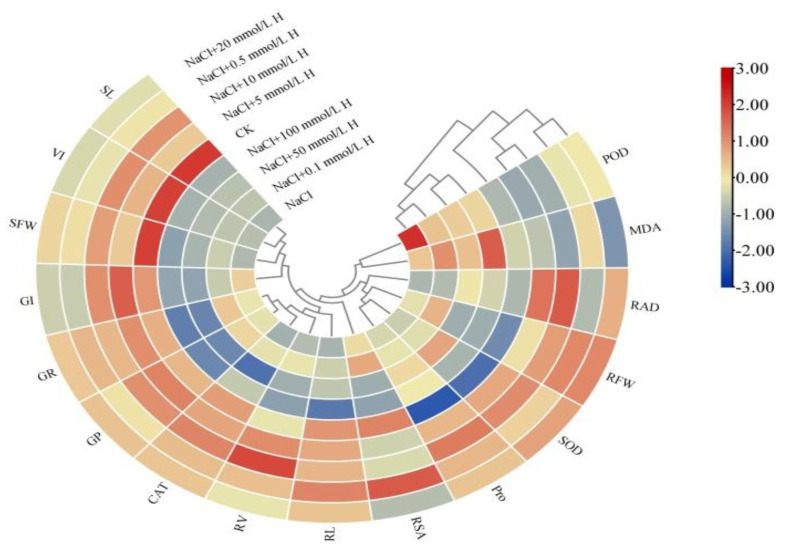
Hierarchical cluster analysis among various salt tolerance indices under different treatments. CK, control treatment (only water); NaCl, 50 mmol L^−1^ NaCl treatment; NaCl + xxx H, treatments of standard NaCl + respective H_2_O_2_ concentration.

The clustering heat map indicates that the high values of all parameters, except POD and MDA, appeared in the 0.5–20 mmol L^−^^1^ H_2_O_2_ treatments (Figure 5). Under NaCl stress, the growth and material accumulation of *F. tataricum* seedlings above and below ground were reduced (the smaller value) (Figure 5). The germination characteristics and morphological characteristics of *F. tataricum* seedlings above and below ground were weakened (the smaller value) by treatments of 50 mmol L^−^^1^ H_2_O_2_ or higher. All the salt tolerance indices, except POD, MDA, and Pro, were reduced (the smaller value) under the NaCl + 100 mmol L^−^^1^ H_2_O_2_ treatment (Figure 5). The maximum values of the above parameters were all reflected in seedlings in the CK, NaCl + 5 mmol L^−^^1^ H_2_O_2_, and NaCl + 10 mmol L^−^^1^ H_2_O_2_ treatments (Figure 5).

**Figure 6:**
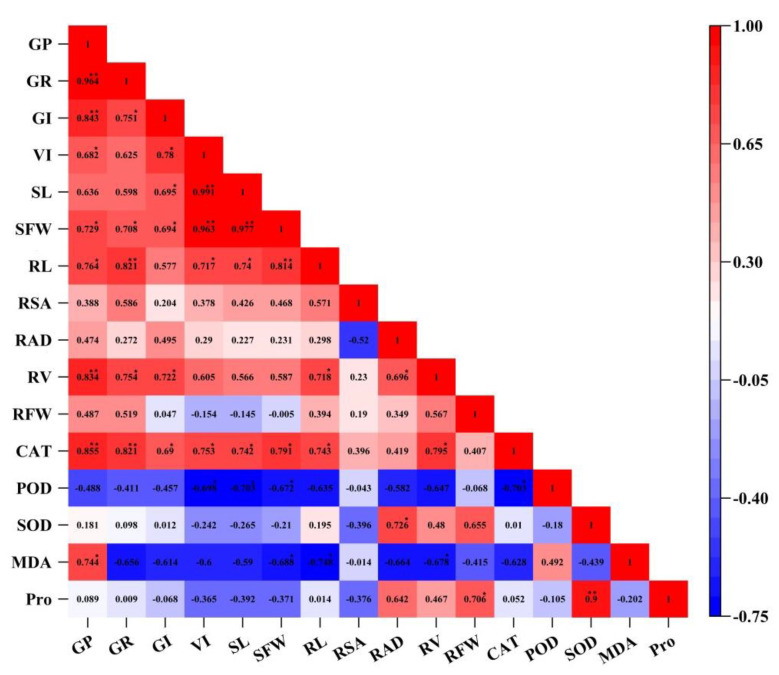
Correlation matrix between all indices. GP, germination potential; GR, germination rate; GI, germination index; VI, vigor index; SL, shoot length; SFW, shoot fresh weight; RL, root length; RSA, root surface area; RAD, root average diameter; RV, root volume; RFW, root fresh weight; CAT, catalase; SOD, superoxide dismutase; POD, peroxidase; MDA, malondialdehyde; Pro, proline. Numbers represent the Pearson correlation statistics. ** denotes significance at *p* < 0.01; * denotes significance at *p* < 0.05.

Correlation analysis indicates whether there is any dependency between the parameters; furthermore, it reveals the strength of the relationship, if any. As shown in Figure 6, SL, VI, and SFW shared a strong, positive correlation (*p <* 0.01). The strongest correlation was between SL and SFW (r = 0.997; *p <* 0.01) (Figure 6). GI was positively correlated with SL, VI, and SFW (*p <* 0.05) (Figure 6); GR shared a strong, positive correlation with GP, CAT, RV, and RL (*p <* 0.01) (Figure 6); and the maximum correlation coefficient was 0.964 (Figure 6). GP shared a strong, positive correlation with CAT and RV (*p <* 0.01); as well as RL (*p <* 0.05) (Figure 6). MDA and POD were negatively correlated with most indices, while they were positively correlated with one another (r = 0.492) (Figure 6). Through the cluster analysis and correlation analysis, the results of the two were consistent (Figure 5 and Figure 6).

### 2.8. Screening Biological Indicators of the H_2_O_2_ Pretreatment and NaCl Stress

#### 2.8.1. Principal Component Analysis

The Origin 2019b Software was used to conduct a principal component analysis (PCA) of the 16 single indices of *F. tataricum* germination and salt tolerance. The eigenvalues of the first three components (PC1, PC2, PC3) were 8.625, 3.795, and 1.726, respectively (Table 3). Their contribution rates were 53.905%, 23.716%, and 10.790%, respectively, and their cumulative contribution was 88.411% (Table 3). As such, PC1, PC2, and PC3 accurately represent all the data contained in the original 16 indices (Table 3).

The eigenvector matrix reflects the load of each index on each principal component. In PC1, GP, CAT, RL, SFW, GR, RV, VI, SL and GI had higher loads and were the main factors of this principal component (Table 3). Thus, PC1 mainly represented the germination characteristics, morphological characteristics, and material synthesis and accumulation of *F. tataricum* seeds (Table 3). In PC2, Pro and SOD were the main factors and had relatively large loads of 0.962 and 0.942, respectively (Table 3). Thus, PC2 mainly represented the antioxidant enzyme activity and osmotic adjustment substances of the seedlings (Table 3). RSA and RAD were the main factors of PC3, indicating that this principle component mainly represented the morphological characteristics and material synthesis and accumulation of the seedlings underground. As such, GP, CAT, RL, SFW, GR, RV, VI, SL, and GI were used as first-level indicators for evaluating salt tolerance of *F. tataricum* seeds treated with different concentrations of H_2_O_2_; Pro and SOD were used as second-level indicators; RSA and RAD were used as third level indicators (Table 3). In summary, 13 indices could be used as comprehensive indicators for evaluating the effects of pretreatment with different H_2_O_2_ concentrations on the germination of *F. tataricum* seeds under NaCl stress (Table 3).

#### 2.8.2. Stepwise Regression Analysis

A mathematical model for the evaluation of salt tolerance was established through stepwise regression. The comprehensive evaluation D value was the dependent variable, and each index value was used as an independent variable in the stepwise regression analysis. The optimal regression equation was set up with the coefficient of determination R^2^ = 1.000, and variance F = 118,710.98.

Y = −0.008X_1_ + 0.241X_2_ + 0.062X_3_ + 0.034X_4_ + 0.048X_5_ − 0.058X_6_ + 0.327,

where Y is the predicted comprehensive evaluation value of *F. tataricum* seeds under salt stress with different H_2_O_2_ pretreatment concentrations; X_1_–X_6_ represent GP, SFW, RSA, RAD, CAT, and SOD, respectively. The correlation coefficient R = 1.000 (P = 0.002) between the predicted salt tolerance value, Y, and the salt tolerance evaluation value, D, of *F. tataricum* seeds, indicates that this equation is accurate and has a high predictive capability. Furthermore, it indicates that the indices used are key to evaluating the influence of different H_2_O_2_ concentrations on *F. tataricum* seeds under NaCl stress.

## 3. Discussion

### 3.1. Salt Stress Adversely Affects Seed Germination of F. Tataricum

Soil salinization is just one of the negative environmental impacts experienced globally as industries strive to increase crop production and yields [18]. Seed germination is the initial and most critical stage in the crop life cycle, and its germination is severely affected by salt stress. As evidenced by the preliminary experiment of this study (Table 1), in which 50 mmol L^−1^ NaCl significantly reduced the GP, GR, and GI of *F. tataricum* seeds, and significantly inhibited their VI and SL, by more than 75.0% (Table 1).

*F. tataricum* seeds are clearly adversely affected by salt stress, as their GP, GR, GI, VI, SL, SFW, RL, RSA, RAD, and RV all decreased significantly under NaCl treatment alone (Figure 1, Figure 2, Figure 3 and Figure 4, Table 2). High extracellular NaCl concentrations create a water potential gradient that draws water from the inside of the cell to the outside. Plants frequently respond by closing their stomata to reduce cell transpiration and prevent cells from absorbing water from the outside [19]. Interestingly, this study found that the RFW of *F. tataricum* seeds increased slightly in a 50 mmol L^−1^ NaCl environment, which might be related to its adaptive strengths as a more tolerant variety (Table 2). *F. tataricum* is better adapted to unfavorable environments such as soils with high salinity and alkalinity and is able to better utilize the resources available in these adverse conditions. It can increase its material accumulation, transportation, and distribution to improve its survival capability. In nature, high salinity environments have many adverse effects on crops, causing the increase and accumulation of intracellular and intercellular antioxidant enzymes, membrane lipid peroxidation, and osmotic adjustment substances [20], protecting cell structure and improving self-resistance.

### 3.2. H_2_O_2_ Pretreatment Alleviates the Negative Effects of NaCl Stress on Seed Germination in F. Tataricum

Hydrogen peroxide (H_2_O_2_) is an exogenous signal substance involved in plant growth regulation and plays a crucial role in their physiological response to adversity. There were some reports on the use of H_2_O_2_ to improve seed germination. For example, H_2_O_2_ application at suitable concentrations alleviated the inhibition of pistachio seed germination under salt stress [21]; reduced the oxidative damage to rape seedlings under low-temperature conditions [9]; and enhanced the growth of wheat and the efficiency of its antioxidant defense systems under drought stress [22]. Due to differences in crop varieties and treatment methods, the optimal concentration at which exogenous H_2_O_2_ should be applied to alleviate NaCl stress is also diverse.

In this study, the germination of *F. tataricum* seeds under NaCl stress was distinctly improved by treatments of different H_2_O_2_ concentrations (Figure 1). Furthermore, the growth of *F. tataricum* seedlings was enhanced significantly (Figure 2, Table 2), the amount of active oxygen was increased, and the accumulation of osmotic adjustment substances was promoted (Figure 3 and Figure 4). Under NaCl stress and increasing H_2_O_2_ concentrations, all indices (except for POD and MDA) increased first and then decreased (Figure 1A–D, Figure 2A,B, Table 2, Figure 3A,B and Figure 4B). SL and RL increased as the H_2_O_2_ concentration increased from 0.1–50 mmol L^−1^, indicating that concentrations of H_2_O_2_ in that range promoted the growth of *F. tataricum* seedlings aboveground and underground (Figure 2A, Table 2). In treatments of 0.1–20 mmol L^−1^ H_2_O_2_, GP, GR, and VI of *F. tataricum* seeds were alleviated (Figure 1A–D); SFW, RV, RFW, and CAT were increased (Figure 2B, Table 2, Figure 3A); and the content of MDA was reduced (Figure 4A). These results indicate that seed vigor was enhanced by treatment within this concentration range. Furthermore, the material accumulation of seedlings was promoted, and the oxidative damage and membrane damage caused by NaCl stress was reduced. POD activity first decreased and then increased with increasing H_2_O_2_ treatment concentration (Figure 3C). One explanation may be that under NaCl stress, peroxisomes were inhibited through redox reactions in cell metabolism during the germination of *F. tataricum* seeds. This subsequently limits the mitigation of NaCl-associated toxic effects by hydrogen peroxide, oxidized phenols and other substances. Another explanation may be that during the germination period of *F. tataricum*, POD activity in its tissues is weak, and the impact of 50 mmol L^−1^ NaCl was not strong enough to cause a significant increase in this enzyme’s activity (Figure 3C).

Furthermore, pretreatment with H_2_O_2_ can alleviate the negative effects of NaCl on the germination characteristics of *F. tataricum* (Figure 1A–D); promote the growth and material accumulation of above-ground and underground parts of the germinated seeds (Figure 2A,B, Table 2); increase the antioxidant enzyme activity and osmotic adjustment substance content in the seedlings (Figure 3A,B and Figure 4B); and reduce the peroxidation of the cell membrane (Figure 4A). However, although low H_2_O_2_ concentrations promote seed germination, high H_2_O_2_ concentrations may poison seeds. As the concentration of H_2_O_2_ is increased, its mitigation effect on NaCl stress is first enhanced and then weakened. Our results suggest that pretreatment with H_2_O_2_ of concentration 5–10 mmol L^−1^ has the best mitigation effect.

### 3.3. Analyses Reveal Six Key Indices to Evaluate the Salt Tolerance of F. Tataricum

Salt tolerance in *F. tataricum* is a complex trait. Researchers have used a variety of methods to evaluate and screen traits that indicate salt tolerance in crop varieties. In this study, a hierarchical cluster analysis and correlation analysis were used to investigate the correlation between various indicators of NaCl stress and the effects of all treatments (Figure 5 and Figure 6). The maximum values of most indicators resulted from the CK, NaCl + 5 mmol L^−1^ H_2_O_2_, and NaCl + 10 mmol L^−1^ H_2_O_2_ treatments (Figure 5). Correlation analysis showed that there was a significant positive correlation among germination parameters, among above-ground indices, and underground indices; and that enzymatic activity was correlated positively or negatively among themselves (Figure 6). When looking at the results of both the cluster analysis and the correlation analysis, the significant relationship between treatments, agronomic traits, and physiological traits can be seen clearly (Figure 5 and Figure 6). Through PCA, this study obtained three new independent principal components with a cumulative contribution rate of 88.411% (Table 3). Concurrently, stepwise regression was used to establish the optimal regression equation for predicting the salt tolerance between the comprehensive evaluation D value and various salt tolerance indicators under H_2_O_2_ seed soaking and NaCl stress. PCA and correlation analysis revealed six key indicators that had a significant impact on the salt tolerance characteristics of *F. tataricum*. These were GP, SFW, RSA, RAD, CAT, and SOD, respectively. Our results suggest that treatment of 5–10 mmol L^−1^ H_2_O_2_ could enhance seed vigor, promote seedling growth, and increase enzymatic activity to effectively alleviate the toxic effects, oxidative damage, and osmotic imbalance caused by NaCl stress (Figure 5 and Figure 6, Table 3).

## 4. Material and Methods

### 4.1. Plant Material

Seeds from the salt-sensitive, “Chuanqiao No. 2” variety of *F. tataricum* were used in this study. They were provided by the Alpine Crops Research Station (102°20′ E and 27°96′ N) of the Xichang Institute of Agricultural Sciences, Liangshan Prefecture, Sichuan Province, China.

### 4.2. Preliminary Seed Sensitivity Experiments Using NaCl

There were five NaCl treatments in this preliminary experiment with 50 *F. tataricum* seeds used in each. The treatments were replicated three times. Healthy *F. tataricum* seeds were carefully selected for uniform size and full grain. The seeds were soaked in distilled water for 12 h; and thereafter, 50 seeds were laid evenly on two layers of quantitative filter paper (9 × 9 cm) in each sterilized petri dish (90 × 90 mm) with different concentrations of NaCl solution (0, 50, 100, 150, and 200 mmol L^−1^). All Petri dishes were placed in a dark incubator room at 22 ± 3 °C for seven days. During this period the germination rate was recorded at 24-h intervals and the NaCl solution was replenished regularly. After seven days, the germination potential, germination rate, germination index, and vigor index were calculated, and the shoot length was determined. The NaCl solution of 50 mmol L^−1^ was selected to induce seed stress in this study because this concentration has a significant inhibitory effect on the vigor index and shoot length of seeds.

### 4.3. Seed Soaking Treatments

This experiment was conducted in the laboratory of the College of Agriculture, Guizhou University, Guiyang, China (26°46′ N and 106°67′ E) in April 2021. Disease-free *F. tataricum* seeds were carefully selected for uniform size and full grain. They were washed with distilled water, disinfected by soaking in 1% NaClO solution for 10 min, rinsed again with distilled water, and then gently blotted to remove any surface moisture. The Hydrogen peroxide (H_2_O_2_) used was analytically pure.

There were nine treatments in this experiment, comprising one control, one NaCl treatment, and seven H_2_O_2_ + NaCl treatments, with 50 *F. tataricum* seeds used in each. These treatments were replicated three times and the germination parameters of the seeds were evaluated. A further four replications were conducted to investigate the effect on physiological indicators. Distilled water was used as the control (CK). NaCl of 50 mmol L^−1^ was used in the NaCl treatment. For the H_2_O_2_ treatments, seeds were soaked in solutions of 0.1, 0.5, 5, 10, 20, 50, and 100 mmol L^−1^ H_2_O_2_ for 12 h under normal temperature and darkness conditions. Thereafter, the soaked seeds were rinsed 3–5 times with distilled water and any moisture remaining on their surface was absorbed by the filter paper. The seeds from each treatment were laid evenly between two layers of quantitative filter paper (9 × 9 cm) in sterilized Petri dishes (90 × 90 mm), and 5 mL of 50 mmol L^−1^ NaCl solution were added to each. Petri dishes were placed in a dark incubator room at 22 ± 3 °C for seven days. During this period, the germination number of each treatment was recorded once a day and the NaCl solution was replenished regularly. After seven days, the germination potential, germination rate, germination index, and vigor index were calculated; as were the root and shoot growth indices of *F. tataricum* seedlings.

### 4.4. Calculation of Germination Parameters, Seedling Traits, and Physiological Indices

#### 4.4.1. Seed Germination Parameters

The seed germination parameters were determined adopting the procedure given by Agami et al. [23]. The germination standard was considered as the radicle length reaching half of the seed length. Germination potential (GP), germination rate (GR), germination index (GI) and vigor index (VI) were calculated using the following equations:

GP = G_3_/N × 100%

GR = G_7_/N × 100%

GI = ∑(G_t_/D_t_)

VI = GI × SL

where G_3_ is the number of germinated seeds on the 3rd day of cultivation; G_7_ is the number of germinated seeds on the 7th day of cultivation; N is the total number of seeds in each treatment (50); G_t_ is the number of seeds germinated at time t; D_t_ is the number of seeds placed in the seedling bed; SL is the average shoot length.

#### 4.4.2. Seedling Traits

After the germination experiment, five seedlings that met the germination standard were randomly selected from each treatment, and their shoots and roots were removed using scissors. Shoot lengths (SL) were measured, averaged, and expressed in cm. Shoot fresh weight (SFW) and root fresh weight (RFW) were measured with an electronic balance; the average values were expressed in g. WinRHIZO Root Analyzer (where it is produced by Guangzhou Hangxin Scientific Instrument Co., Ltd., Guangzhou, China) was used to measure root traits, including root length (RL), root surface area (RSA), root average diameter (RAD), and root volume (RV).

#### 4.4.3. Physiological Indices

Physiological indices were measured by rapid methods as suggested by Lu et al. [24] and the instructions of the BOXBIO kit. Catalase (CAT) and proline (Pro) were run through an ultraviolet spectrophotometer (where it is produced by Unico Instrument Co., Ltd., Shanghai, China) to determine the absorbance at 240 nm and 520 nm, respectively. Superoxide dismutase (SOD) and peroxidase (POD) were measured using a microplate reader (where it is produced by Chengdu Baile Technology Co., Ltd., Chengdu, China) at 560 nm and 470 nm, respectively; and malondialdehyde (MDA) was measured at 450, 532, and 600 nm, respectively.

### 4.5. Statistical Analysis

Microsoft Excel 2010 was used for data input, processing of the original data, and basic statistical analysis. SPSS software version 26 was used to perform single-factor analysis of variance, Pearson correlation analysis, and stepwise regression analysis to screen the salt tolerance index of germination of *F. tataricum* seeds for each germination parameter and physiological indicator. Duncan’s multiple range test (*p <* 0.05) was used to measure the significance of differences. The results are expressed as means ± standard deviations. Variance analysis charts, correlation heat map, and principal component analysis were created using the Origin 2019b Software. The cluster heat map was created using Tbtools.

## 5. Conclusions

In this study, 5–10 mmol L^−^^1^ H_2_O_2_ effectively enhanced the vigor of “Chuanqiao-2” *F. tataricum* seeds; promoted their germination and growth; increased their enzymatic activity; and efficiently alleviated the toxic effects, oxidative damage, and osmotic imbalances caused by salt stress. Germination potential, shoot fresh weight, root surface area, root average diameter, catalase activity, and superoxide dismutase activity were the six key indicators that can be used to assess the impact of salt stress in *F. tataricum*. In this study, only the germination of *F. tataricum* seeds, the phenotype estimate of the shoots and roots of the seedlings, and the determination of related enzyme activities were involved, but the study of gene expression and molecular mechanisms was not researched. Therefore, it is necessary to study the effect of H_2_O_2_ on *F. tataricum* under salt stress.

## Figures and Tables

**Table 1:** Effects of different NaCl concentrations on germination parameters of *F. tataricum* seeds.

Treatment	Germination Potential (%)	Germination Rate (%)	Germination Index	Vigor Index	Shoot Length (cm)
0 mmol L^−^^1^ NaCl	88.0 ± 0.040 c	97.3 ± 0.031 b	99.569 ± 10.106 c	715.097 ± 41.106 d	7.211 ± 0.496 d
50 mmol L^−^^1^ NaCl	82.7 ± 0.058 bc	92.0 ± 0.053 ab	93.260 ± 8.811 c	164.676 ± 23.760 c	1.760 ± 0.092 c
100 mmol L^−^^1^ NaCl	76.7 ± 0.023 bc	91.3 ± 0.012 ab	66.274 ± 1.392 b	84.301 ± 4.980 b	1.272 ± 0.076 b
150 mmol L^−^^1^ NaCl	68.7 ± 0.050 b	86.7 ± 0.046 a	56.496 ± 3.695 b	58.112 ± 4.974 ab	1.031 ± 0.095 ab
200 mmol L^−^^1^ NaCl	47.3 ± 0.147 a	85.3 ± 0.042 a	43.362 ± 6.115 a	34.121 ± 2.830 a	0.792 ± 0.060 a

The data in the table are represented as the mean ± standard deviation, and different lowercase letters in the same column indicate significant differences between treatments at *p <* 0.05.

**Table 2:** Effects of H_2_O_2_ on root growth of *F. tataricum* seedlings under NaCl stress.

Treatment	Root Length (cm)	Root Surface Area (cm^2^)	Root Average Diameter (mm)	Root Volume (cm^3^)	Root Fresh Weight (g)
CK	15.842 ± 1.509 d	1.695 ± 0.196 ef	0.340 ± 0.010 a	0.014 ± 0.002 bc	0.009 ± 0.002 a
NaCl	10.095 ± 1.126 ab	1.035 ± 0.175 d	0.326 ± 0.039 a	0.009 ± 0.003 ab	0.014 ± 0.001 bc
NaCl + 0.1 mmol L^−^^1^ H_2_O_2_	11.455 ± 3.494 abc	1.412 ± 0.436 e	0.392 ± 0.010 ab	0.014 ± 0.004 bc	0.017 ± 0.001 cd
NaCl + 0.5 mmol L^−^^1^ H_2_O_2_	16.473 ± 3.627 d	2.005 ± 0.350 f	0.390 ± 0.024 ab	0.019 ± 0.003 cd	0.019 ± 0.001 d
NaCl + 5 mmol L^−^^1^ H_2_O_2_	15.129 ± 1.431 cd	0.676 ± 0.037 bcd	2.122 ± 0.117 e	0.024 ± 0.005 de	0.015 ± 0.006 bc
NaCl + 10 mmol L^−^^1^ H_2_O_2_	14.634 ± 2.813 cd	0.752 ± 0.165 cd	2.362 ± 0.518 e	0.031 ± 0.009 e	0.018 ± 0.003 cd
NaCl + 20 mmol L^−^^1^ H_2_O_2_	14.127 ± 2.094b cd	0.490 ± 0.0240 abc	1.538 ± 0.075 d	0.014 ± 0.002 bc	0.019 ± 0.002 cd
NaCl + 50 mmol L^−^^1^ H_2_O_2_	11.005 ± 0.712 abc	0.324 ± 0.029 ab	1.017 ± 0.092 c	0.007 ± 0 ab	0.011 ± 0.001 ab
NaCl + 100 mmol L^−^^1^ H_2_O_2_	7.360 ± 1.020 a	0.220 ± 0.027 a	0.691 ± 0.085 b	0.005 ± 0.002 a	0.011 ± 0.002 ab

The data in the table are represented as the mean ± standard deviation, and different lowercase letters in the same column indicate significant differences between treatments at *p* < 0.05.

**Table 3:** Eigenvalues, contribution rates, and eigenvectors for each principal component.

Items		PC1	PC2	PC3
Eigenvalue		8.625	3.795	1.726
Contribution rate (%)		53.905	23.716	10.790
Cumulative contribution ratio (%)		53.905	77.621	88.411
Eigenvector	GP	0.923	0.081	0.197
	CAT	0.908	−0.032	0.084
	RL	0.887	−0.042	0.211
	SFW	0.884	−0.403	−0.117
	GR	0.878	−0.019	0.401
	RV	0.873	0.375	0.048
	VI	0.855	−0.421	−0.273
	SL	0.836	−0.455	−0.236
	GI	0.819	−0.087	−0.164
	MDA	−0.805	−0.272	0.129
	POD	−0.722	−0.046	0.411
	Pro	0.047	0.962	0.074
	SOD	0.162	0.942	−0.021
	RAD	0.524	0.688	−0.492
	RFW RSA	0.352 0.388	0.678 −0.518	0.604 0.710

GP, germination potential; CAT, catalase; RL, root length; SFW, shoot fresh weight; GR, germination rate; RV, root volume; VI, vigor index; SL, shoot length; GI, germination index; MDA, malondialdehyde; POD, peroxidase; Pro, proline; SOD, superoxide dismutase; RAD, root average diameter; RFW, root fresh weight; RSA, root surface area.

## Data Availability

All data are included in the present study.
